# Bacterial Outer Membrane Protein OmpX Regulates β1 Integrin and Epidermal Growth Factor Receptor (EGFR) Involved in Invasion of M-HeLa Cells by *Serratia proteamaculans*

**DOI:** 10.3390/ijms222413246

**Published:** 2021-12-09

**Authors:** Olga Tsaplina, Ekaterina Bozhokina

**Affiliations:** Institute of Cytology, Russian Academy of Sciences, 194064 St. Petersburg, Russia; bozhokina@yahoo.com

**Keywords:** bacterial invasion, outer membrane protein OmpX, *Serratia proteamaculans*, EGFR, β1 integrin, fibronectin

## Abstract

Opportunistic pathogen *Serratia proteamaculans* are able to penetrate the eukaryotic cells. The penetration rate can be regulated by bacterial surface protein OmpX. OmpX family proteins are able to bind to host cell surface to the epidermal growth factor receptor (EGFR) and the extracellular matrix protein fibronectin, whose receptors are in return the α5 β1 integrins. Here we elucidated the involvement of these host cell proteins in *S. proteamaculans* invasion. We have shown that, despite the absence of fibronectin contribution to *S. proteamaculans* invasion, β1 integrin was directly involved in invasion of M-HeLa cells. Herewith β1 integrin was not the only receptor that determines sensitivity of host cells to bacterial invasion. Signal transfer from EGFR was also involved in the penetration of these bacteria into M-HeLa cells. However, M-HeLa cells have not been characterized by large number of these receptors. It turned out that *S. proteamaculans* attachment to the host cell surface resulted in an increment of EGFR and β1 integrin genes expression. Such gene expression increment also caused *Escherichia coli* attachment, transformed with a plasmid encoding OmpX from *S. proteamaculans*. Thus, an OmpX binding to the host cell surface caused an increase in the EGFR and β1 integrin expression involved in *S. proteamaculans* invasion.

## 1. Introduction

Bacterial adhesion and invasion of host cells can determine the degree of bacterial pathogenicity. Tight bindings of microbes to host cells are often a pre-requisite to colonize host surfaces, which could trigger signaling pathways responsible for bacteria uptake or bacterial effectors injection into eukaryotic cells. The bacterial components have evolved a capacity to attach to host adhesion molecules. Host-cell adhesion molecules are cell-surface receptors that mediate cell–to-cell and cell–extracellular-matrix interactions. By binding to host cells adhesion molecules, bacteria act instead of host cell receptors or their ligands and trigger main signaling pathways leading to invasion.

*Serratia* spp. are opportunistic pathogens that can pose a dangerous threat to hospitalized and immunocompromised patients [1]. Previously we have shown that *S. proteamaculans* is capable to penetrate human cells [2]. The invasive activity of *S. proteamaculans* can be regulated by the outer membrane protein OmpX [3] via increasing a bacterial adhesion [4]. The OmpX-family surface proteins are responsible for antibiotic resistance and signal transduction, as well as virulence, killer resistance, adhesion, and invasion [5]. OmpX family proteins inactivation can be lead the invasion rate decrease as a result of adhesion reduction of pathogenic *E. coli* [5] and *Yersinia pestis* [6,7] or without affecting the adhesion of *Salmonella enterica* [8] and *Cronobacter sakazakii* [9].

OmpX family proteins have shown the ability to bind to the .EGFR [10] and extracellular matrix protein fibronectin [7]. Fibronectin binds to α5 and β1 integrins heterodimer on eukaryotic cells surface and trigger an activation of these receptors. β1 integrin and EGFR activation induce recruitment and non-receptor tyrosine kinases Src or/and FAK stimulation [11,12], thereby triggering actin cytoskeleton rearrangement which are necessary for bacterial penetration.

The aim of this work was to determine the role of EGFR, β1 integrin and fibronectin in *S. proteamaculans* invasion. We have shown that signal transfer from EGFR was involved in the penetration of these bacteria into M-HeLa cells. Furthermore, β1 integrin was also involved in the invasion of *S. proteamaculans* vice-verse to fibronectin, which was not involved in this process. It turned out that attachment of *S. proteamaculans* to eukaryotic cell surface resulted in amplification of EGFR and β1 integrin genes expression. Such expression increment of these receptors involved in invasion causes an *S. proteamaculans* OmpX interaction with the host cell surface.

## 2. Materials and Methods

### 2.1. Cell Cultures, Bacterial Strains, and Growth Conditions

The cervical carcinoma M-HeLa cell line was obtained from the “Vertebrate cell culture collection” (Institute of Cytology, St. Petersburg, Russia) supported by the Ministry of Science and Higher Education of the Russian Federation (Agreement № 075-15-2021-683). Cells were grown in αMEM medium contained 1% nonessential amino acids (NEAAs) (Sigma-Aldrich, Taufkirchen, Germany) and 10% fetal bovine serum (Sigma-Aldrich, Taufkirchen, Germany). 

*Serratia proteamaculans* 94 was isolated as described earlier [13]. The recombinant *E. coli* (OmpX), expressing *S. proteamaculans* 94 *OmpX* gene with 6-His at the C-terminus was obtained as described previously [3]. *E. coli* (pET21a) transformed with the plasmid pET21a was used as a control [3].

### 2.2. Adhesion to Fibronectin

Fibronectin 5 μg/mL in PBS solution was added to the well for 45 min at 37 °C. To prevent a nonspecific binding, fibronectin solution was changed to 1% BSA in PBS solution for 30 min. After three washes with PBS solution, bacteria diluted in DMEM medium were added for 2 h at 37 °C. Unattached bacteria were removed by three washes with PBS solution. Then the bacteria attached to the well were detached with washing with trypsin-versene solution. This bacterial suspension was diluted 10 times in LB at 0 °C the required number of times, and aliquots (100 μL) of the resulting suspension were plated on LB (Luria broth) agar.

### 2.3. siRNA Transfection

The expression of host cell proteins was inhibited using siRNA targeting fibronectin (sc-35371), β1 integrin (sc-35674), EGFR (sc-29301) (Santa Cruz, Dallas, TX, USA). Transfection of siRNAs was performed using siRNA Transfection Reagent (sc-29528) as recommended by the manufacturer (Santa Cruz, Dallas, TX, USA). The RNA interference efficiency was controlled by real-time RT-PCR and Western blotting.

### 2.4. Western Blot Analysis

After transfection of siRNAs or incubation with *S. proteamaculans*, cells were incubated with electrophoresis sample buffer (4% SDS, 24% glycerol, 200 mM DTT, 0.01% bromphenol blue, 125 mM Tris-HCl, pH 6.8) for 5 min at 56 °C. Cells were scraped off the plate, followed by a 5 min boiling. The samples were fractionated by SDS-PAGE and transferred to a Hybond ECL membrane according to the manufacturer’s instructions (GE Healthcare, Chalfont Saint Giles, UK). The membrane was incubated with 5% nonfat milk in PBS 40 min to prevent nonspecific binding of antibodies and then incubated with rabbit primary antibodies against EGFR [E235] at a dilution of 1:1000 (Abcam, Cambridge, UK), β1 integrin [EPR16895] at a dilution 1:1000 (Abcam, Cambridge, UK), fibronectin [EP-5] at a dilution 1:1000 (Abcam, Cambridge, UK), GADPH at a dilution 1:1000 (Abcam, Cambridge, UK) at room temperature for 1 h. The membrane was then washed three times with washing buffer (5% nonfat milk, 0.1% Tween 20, PBS) for 10 min, incubated for 2 h with the secondary antibodies (1:20000) against rabbit IgG conjugated with horseradish peroxidase. The blots were washed with washing buffer three times and developed using SuperSignal West FEMTO Chemiluminescent Substrate (ThermoFisher Scientific, Walthan, MA, USA) according to the manufacturer’s recommendations.

### 2.5. Quantitative Invasion Assay

Efficiency of invasion was evaluated by the quantitative invasion assay [3,14]. Cells forming a 50–70% monolayer were transfected with RNA or treated with tyrphostin AG 1478 for 1.5 h before adding bacteria. *S. proteamaculans* were grown in LB medium till the late stationary growth phase until actinase activity on the *S. proteamaculans* extract could be determined [2]. Bacteria were pelleted at 9600 g for 10 min; the pellets were resuspended in DMEM and added to host cells in a fresh portion of DMEM at a ratio of 100 bacteria per cell. After co-cultivating host cells and bacteria at 37 °C in 5% CO_2_ for 2 h unattached bacteria were washed out twice with PBS, and the infected cells were suspended in 0.25% trypsin-versene solution. To quantify the effectiveness of invasion, suspension of the infected cells was incubated in DMEM containing kanamycin to kill extracellular bacteria and then cells were lysed with 1.5% sodium deoxycholate, quickly diluted with cold LB medium and aliquots of the resulting suspension were plated on LB-agar to determine the number of colony forming units (CFU) of intracellular bacteria [3]. The results for each experiment were the average of an assay performed in triplicate and independently repeated three times.

### 2.6. Fluorescence Microscopy

Cells were grown at 37 °C in an atmosphere of 5% CO_2_ on coverslips until a 70–80% monolayer was formed. Bacteria *S. proteamaculans* were grown in LB medium (Sigma-Aldrich, Taufkirchen, Germany) at 30 °C with aeration for 44–48 h until the actinase activity of *S. proteamaculans* extracts could be detected [2]. The bacterial suspension was centrifuged at 9600 g, 8 min. The pellet was resuspended in DMEM medium and added to eukaryotic cells at a ratio of 100 bacteria per cell. The host cells and bacteria were co-cultivated at 37 °C in 5% CO_2_ for 3 h. Cells were washed three times with PBS solution at each staining step. The preparations were fixed with 3.7% formaldehyde solution (Sigma-Aldrich, Taufkirchen, Germany) for 10 min and incubated for 5 min with 0.1% Triton X100, and, then, with 1% bovine serum albumin for 30 min to prevent nonspecific binding of antibodies. Eukaryotic cell receptors were stained with the rabbit primary antibodies against EGFR [E235] at a dilution of 1:500 (Abcam, Cambridge, UK) and the mouse antibodies against β1 integrin [P5D2] at a dilution 1:250 (Abcam, Cambridge, UK). The preparations were incubated for 1 h with secondary antibodies against rabbit or mouse, cross linked with Alexa-488 (Santa Cruz, Dallas, TX, USA) at a dilution of 1:500, and stained with rhodamine-phalloidin for 15 min to visualize the actin cytoskeleton and DAPI for 15 min to visualize DNA of bacteria and epithelial cells. The preparations were analyzed using an Olympus FV3000 microscope (Japan) using a system of lasers with wavelengths of 405 (blue fluorescence), 488 (green fluorescence), and 561 nm (red fluorescence).

### 2.7. Real-Time RT-PCR

Total RNA was extracted from M-HeLa cells using Dia-M Extraction Kit according to the manufacturer’s instructions (Dia-M, Moscow, Russia). Reverse transcription was performed with the RevertAid First Strand cDNA Synthesis Kit (Thermo Fisher Scientific, Walthan, MA, USA), and the resulting cDNA was diluted in H_2_O to 20 ng/mL. Amplification was conducted in a 20 µL of diluted cDNA with the SYBR Green reagents (Syntol, Moscow, Russia) using CFX96 Touch Real-Time PCR machine (Bio-Rad, Irvine, CA, USA). The steps included initial denaturation 95 C for 30s, and 40 cycles of 95 °C for 5 s, 58 or 60 °C for 30 s and 72C for 15 s. Each sample was run in triplicate. Target gene expression was normalized to the expression of a cellular housekeeping gene, β-actin or GADPH, and calculated using the 2-∆∆CT method. Gene-specific primer pairs (Evrogen, Moscow, Russia) designed using BLAST-primer software and annealing temperatures used for real-time PCR are listed in Table 1:

### 2.8. Statistical Analysis

Each quantitative experiment was repeated at least three times. Data were analyzed statistically using one-way analysis of variance (ANOVA) with Excel Data Analysis Pack. A difference was considered significant at the *p* < 0.05 level.

## 3. Results

### 3.1. Fibronectin in S. proteamaculans Invasion

**Figure 1:**
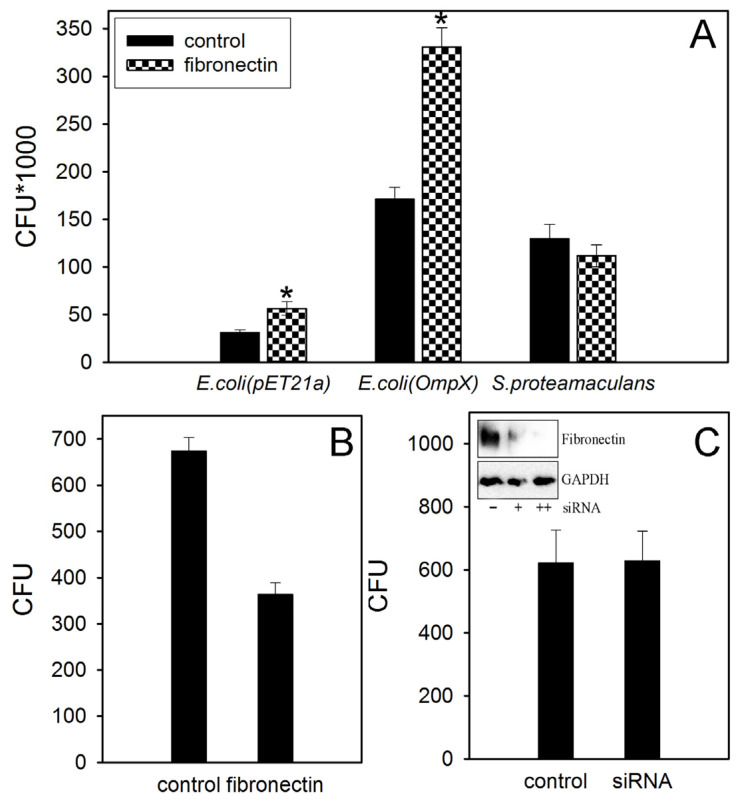
Involvement of fibronectin in *S. proteamaculans* invasion. (**A**) Adhesion of *E. coli* (pET21a) not carrying *S. proteamaculans* OmpX or *S. proteamaculans* and *E. coli* (OmpX) encoding *S. proteamaculans OmpX* gene to plates coated with fibronectin. Control, plate uncoated with fibronectin. (**B**) The effect of pre-incubation of *S. proteamaculans* with fibronectin for 1 h at 37 °C on the intensity of bacterial invasion of M-HeLa cells. Control, untreated bacteria. Values are expressed as mean S.D. (error bars). A difference was considered significant at the * *p* < 0.05 level. (**C**) Effect of treating M-HeLa cells with siRNA targeting fibronectin on cell sensitivity to *S. proteamaculans* invasion. Control, M-HeLa cells transfected with siRNA containing scrambled nucleotide sequence. The insert shows the total amount of fibronectin and internal control GAPDH in untreated M-HeLa cells (“−”) and pretreating cells with small interfering RNA at the minimum (“+”) and maximum (“++”) concentration according to the manufacturer’s protocol. Values are expressed as mean S.D. (error bars). A difference was considered significant at the *p* < 0.05 level. Pretreating cells with a maximum concentration of small interfering RNA reduced the fibronectin expression by 29% (Figure S1A).

Previously, we have shown that OmpX can stimulate *Serratia* invasion [3]. The homologous OmpX protein can interact with fibronectin on the host cell surface [7]. Fibronectin is an extracellular matrix protein that is involved in cell adhesion, growth, migration and differentiation. In order to determine whether the OmpX from *S. proteamaculans* binds to fibronectin, we evaluated the effect of coating plates with fibronectin on bacterial adhesion intensity. We showed that transformation of *E. coli* with a plasmid carrying the *S. proteamaculans OmpX* gene results to a six-fold increase in adhesion to fibronectin (Figure 1A). However, coating the plates with fibronectin did not increase the adhesion of *S. proteamaculans* (Figure 1A). Moreover, pre-incubation with purified fibronectin halved the intensity of *S. proteamaculans* invasion (Figure 1B). Excess fibronectin appears to interfere with the binding of bacteria to specific receptors on the host cell. This allowed us to assume that fibronectin was not involved in this bacteria invasion. To test this assumption, we used small interfering RNA targeting fibronectin. Indeed, pretreating cells with this siRNA did not affect the sensitivity of M-HeLa cells to bacteria (Figure 1C). Thus, fibronectin does not seem to be involved in *S. proteamaculans* penetration into M-HeLa cells.

### 3.2. β1 Integrin in S. proteamaculans Invasion

**Figure 2:**
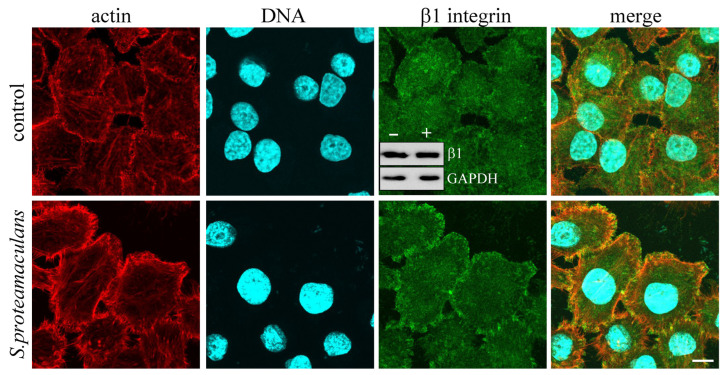
Distribution of β1 integrin in M-HeLa cells as a result of *S. proteamaculans* invasion. Cells were incubated with bacteria for 3 h. Control-uninfected M-HeLa cells. Cytoskeleton was stained with rhodamine-phalloidin; β1 integrin was stained with antibodies; DNA was stained with DAPI. Scale bar: 10 μm. The insert shows a total amount of β1 integrin and internal control GAPDH in M-HeLa cells incubated for 3 h in absence (“−”) or presence (“+”) of *S. proteamaculans*.

**Figure 3:**
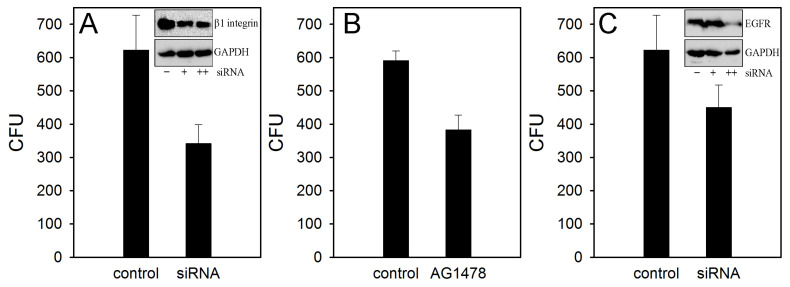
Involvement of host cell receptors in *S. proteamaculans* invasion. (**A**) Effect of treating M-HeLa cells with siRNA targeting β1 integrin on cell sensitivity to *S. proteamaculans* invasion. Control-M-HeLa cells transfected with siRNA containing scrambled nucleotide sequence. The insert shows the total amount of β1 integrin and internal control GAPDH in untreated M-HeLa cells (“−”) and pretreating cells with small interfering RNA at the minimum (“+”) and maximum (“++”) concentration according to the manufacturer’s protocol. Values are expressed as mean S.D. (error bars). A difference was considered significant at the *p* < 0.05 level. Pretreating cells with a maximum concentration of small interfering RNA reduced the β1 integrin expression by 46% (Figure S1B). (**B**) Effect of pre-incubation of M-HeLa cells with tyrphostin AG 1478 for 1.5 h on cell sensitivity to invasion. Control-untreated M-HeLa cells. Values are expressed as mean S.D. (error bars). A difference was considered significant at the *p* < 0.05 level. (**C**) Effect of treating M-HeLa cells with siRNA targeting EGFR on cell sensitivity to *S. proteamaculans* invasion. Control-M-HeLa cells transfected with siRNA containing scrambled nucleotide sequence. The insert shows the total amount of EGFR and internal control GAPDH in untreated M-HeLa cells (“−”) and pretreating cells with small interfering RNA at the minimum (“+”) and maximum (“++”) concentration according to the manufacturer’s protocol. Values are expressed as mean S.D. (error bars). A difference was considered significant at the *p* < 0.05 level. Pretreating cells with a maximum concentration of small interfering RNA reduced the EGFR expression by 28% (Figure S1C).

We have previously shown that in response to bacterial penetration, there were an β1 and α5 integrins accumulation on the surface of infected cell [15]. Heterodimer of α5 and β1 integrins forms a fibronectin receptor in host cells. Therefore, despite the absence of fibronectin effect on *S. proteamaculans* invasion, we evaluated the contribution of β1 integrin in this process. In response to *S. proteamaculans* infection, β1 integrin was transferred from cytoplasm to the surface of infected cell and accumulates the infected cells perimeter (Figure 2). At the same time, a total amount of β1 integrin in M-HeLa cells have not change during bacterial infection (Figure 2, insert). To determine a necessity of β1 integrin in bacterial invasion, we have used small interfering RNA. The sensitivity of M-HeLa cells pre-treated with siRNA targeting β1 to *S. proteamaculans* was halved (Figure 3A). Thus, despite a fact that fibronectin have not participate in the *S. proteamaculans* invasion; our data indicate that β1 integrin was directly involved in this process.

### 3.3. EGFR in S. proteamaculans Invasion

**Figure 4:**
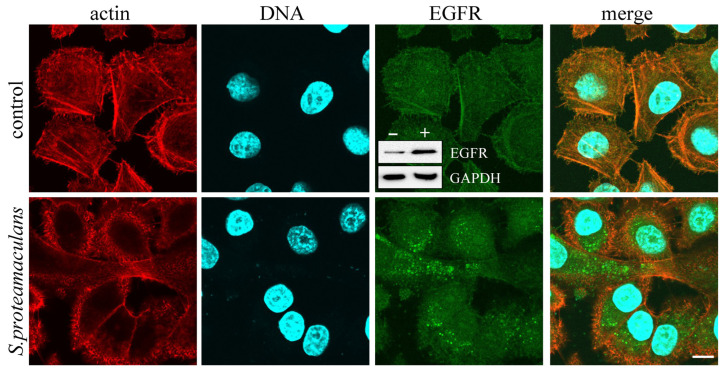
Distribution of EGFR in M-HeLa cells as a result of *S. proteamaculans* invasion. Cells were incubated with bacteria for 3 h. Control-uninfected M-HeLa cells. Cytoskeleton was stained with rhodamine-phalloidin; β1 integrin was stained with antibodies; DNA was stained with DAPI. Scale bar: 10 μm. The insert shows the total amount of EGFR and internal control GAPDH in M-HeLa cells incubated for 3 h in the absence (“−”) or presence (“+”) of *S. proteamaculans.*

In the control cells, EGFRs were co-localized with actin fibrils. In response to infection with *S. proteamaculans*, EGFRs were accumulated in epithelial cells cytoplasm [15]. The EGFR accumulation in infected A549 cells was similar to accumulation in endosomes when the receptor binds to EGF [15]. When M-HeLa cells were infected with *S. proteamaculans*, individual cells with a similar redistribution of EGFR were found (Figure 4). At the same time, a total amount of EGFR in cells was increased during infection (Figure 4, insert). In order to determine whether the signal from EGFR was involved in *S. proteamaculans* invasion, we used the tyrphostin AG 1478 inhibitor. Tyrphostin AG 1478 inhibits ligand binding-induced phosphorylation of EGFR and EGFR signal transmission. Cells treatment with tyrphostin AG 1478 reduced M-HeLa cells sensitivity to bacterial invasion by 30% (Figure 3B). The same quantitative effect on M-HeLa cells sensitivity to *S. proteamaculans* was caused by a pretreating the host cells with small interfering RNA targeting EGFR (Figure 3C). Thus, EGFR role in *S. proteamaculans* invasion appeared to be a signal transduction from the cell surface.

### 3.4. Effect of Serratia Infection on Expression of Host Cell Proteins

**Figure 5:**
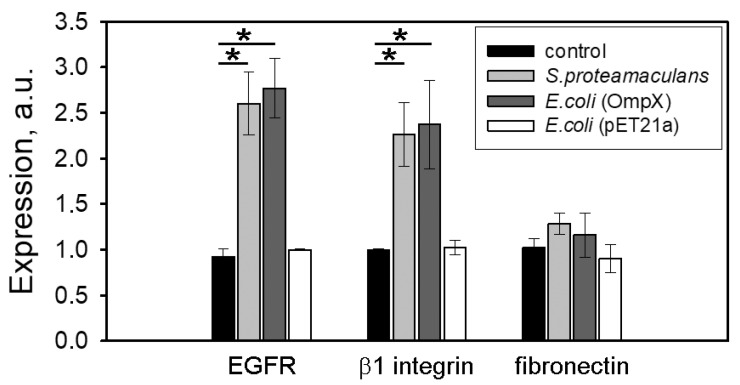
Effect of bacterial infection on proteins expression in the host cell. Using real-time RT-PCR, expression levels of EGFR, β1 integrin and fibronectin were determined after 2 h of incubation with *S. proteamaculans*, *E. coli* (OmpX) encoding *OmpX* gene from *S. proteamaculans* or *E. coli* (pET21a) not carrying OmpX gene from *S. proteamaculans*. Control-uninfected M-HeLa cells. GADPH and β-actin served as an internal control. Values are expressed as mean S.D. (error bars). A difference was considered significant at the * *p* < 0.05 level.

We have shown that *S. proteamaculans* use β1 integrins and EGFR to enter M-HeLa cells. However, the number of these receptors on M-HeLa cells surface could be limited (Figure 2 and Figure 4). Therefore, using real-time RT-PCR, we evaluated the effect of infection on the expression of the host cell proteins genes. We showed that infection with the *S. proteamaculans* leads to an increase in β1 integrin and EGFR expression by 2–2.5 times (Figure 5). At the same time, infection practically does not affect the fibronectin expression. The same effect on proteins expression in M-HeLa cells was caused by infection with *E. coli* (OmpX) transformed with the plasmid carrying the *OmpX* gene. At the same time, control *E. coli* during infection did not affect the expression of host cell receptors. Thus, the OmpX binding to the host cell surface causes an increase in the expression of EGFR and β1 integrin, which were involved in *S. proteamaculans* invasion.

## 4. Discussion

Bacterial proteins that mediate host cell adhesion and trigger internalization into eukaryotic cell could target a variety of surface receptors, and most of these receptors belong to cell adhesion molecules group. Different bacterial proteins recognize many host-cell surface elements, including extracellular matrix components, which can serve as a linkage between bacterium and host cell receptor. Adhesive glycoproteins such as fibronectin—which can be present as secreted or plasma membrane-associated molecules—are recognized by many different species of bacterial pathogens. Each bacterium can use different receptors which recognize specific fibronectin domains, mostly the N-terminal and the central cell-binding domains [16]. One bacterium can synthesize a set of surface proteins that interact with fibronectin; at least 11 fibronectin-binding proteins have been identified in *Streptococcus pyogenes* [17]. Among the Gramm-negative bacteria, two large protein families–Omp family porins and YadA-like autotransporter proteins are frequently bind fibronectin [18]. OmpX from *S. proteamaculance* also interacts with fibronectin. Despite this, we have shown that fibronectin was not involved in *S. proteamaculance* invasion of M-HeLa cells.

Fibronectin attached to bacterial surface proteins interacts with α5 β1 integrins [19]. This interaction leads to the cytoskeleton actin rearrangement in host cells and the capture of invading bacteria. Bacterial proteins can interact with integrins not only through fibronectin, but also directly [19]. The *Yersinia pseudotuberculosis* invasin promotes bacterial entry by binding to host cell integrins with higher affinity than to fibronectin [20]. We have shown that β1 integrin was involved in the *S. proteamaculance* invasion and co-incubation with these bacteria leads to accumulation of this receptor along infected cell perimeter. Binding of bacterial proteins to host-cell integrins can lead to integrin clusters formation, which triggers remodeling of the actin cytoskeleton and promotes bacterial internalization into epithelial cells by “zipper” invasion mechanism [21].

Integrin clustering in lipid rafts leads to recruitment and activation of tyrosine kinases FAK and Src [22,23,24], which target small Rho GTPases, thereby controlling actin cytoskeleton rearrangements [25]. Furthermore, phosphorylation of FAK and Src triggers a cascade of signals resulting in the formation of protein complexes leading to activation of other signaling factors as EGFR, followed by activation of small Rho GTPase [24]. This signaling potentially causes localized actin and/or microtubule rearrangements at the site of bacterial entry, resulting in bacterial uptake. We have shown that EGFR is also involved in the *S. proteamaculance* invasion and that inhibition of signal transduction from the EGFR decreases a sensitivity of host-cells to bacteria.

Thus, we have shown that both β1 integrin and EGFR were involved in *S. proteamaculans* invasion of M-HeLa cells. However, a set of surface receptors in the cells of different tissues may differ, while the sensitivity of different cells to *Serratia* remains practically the same level [26,27,28]. In this work, we showed that host-cells infection with *S. proteamaculans* leads to an increase of β1 integrin and EGFR genes expression, and this effect was caused by OmpX interaction with host cells. Our results for the first time shown that contact of bacterial proteins with host cell could cause the accumulation of receptors needed for *S. proteamaculans* invasion.

## Figures and Tables

**Table 1:** Gene-specific primer pairs and their annealing temperatures.

Target Gene	Primer Sequences	Annealing Temperatures
EGFR	Forward 5’-GTGCAGCTTCAGGACCACAA-3′ Reverse 5′-AAATGCATGTGTCGAATATCTTGAG-3′	60 °C
β1 integrin	Forward 5’-GACGCCGCGCGGAAAAG-3′ Reverse 5′-ATCTGGAGGGCAACCCTTCT-3′	58 °C
fibronectin	Forward 5’-CCCATCACAGGGTACAGAATAG-3′ Reverse 5-CGGTGTTGTAAGGTGGAATAGA-3′	58 °C
β-actin	Forward 5’-CACCAACTGGGACGACAT-3’ Reverse 5’-ACAGCCTGGATAGCAACG-3’	58 °C
GADPH	Forward 5’-GGATTTGGTCGTATTGGG-3’ Reverse 5’-GGAAGATGGTGATGGGATT-3’	58 °C

## Data Availability

Not applicable.

## Supplementary Materials

The following are available online at https://www.mdpi.com/article/10.3390/ijms222413246/s1.
