# Supplementary material for: Bacterial Outer Membrane Protein OmpX Regulates β1 Integrin and Epidermal Growth Factor Receptor (EGFR) Involved in Invasion of M-HeLa Cells by Serratia proteamaculans
Source: Int J Mol Sci. 2021 Dec 9;22(24):13246. doi: 10.3390/ijms222413246 (PMC8703988; doi:10.3390/ijms222413246)
Supplement: Supplementary file 1 [file ijms-22-13246-s001.zip › ijms-1486143-supplementary.pdf]

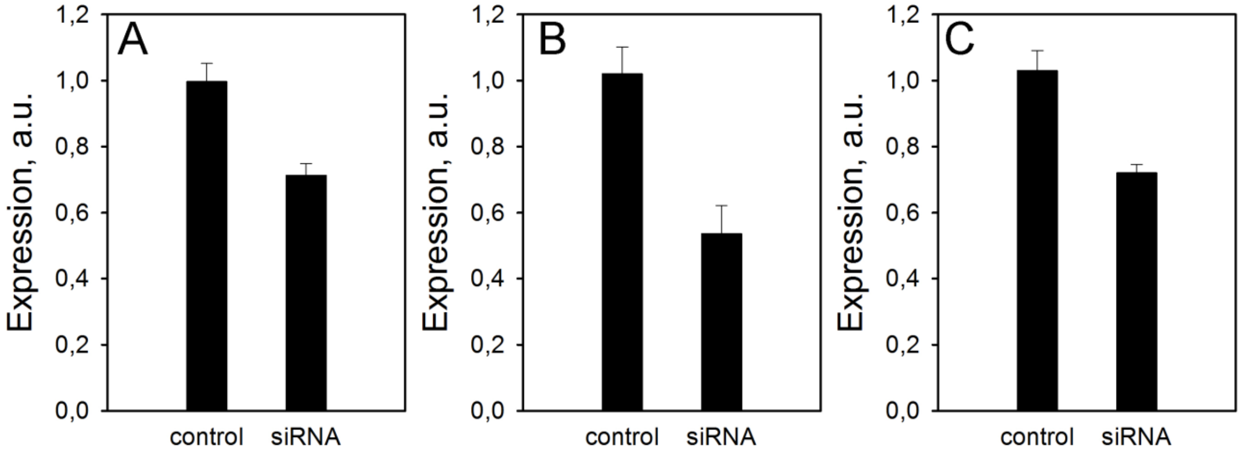

Figure S1. Effect of treating M-HeLa cells with siRNA on proteins expression in the host cell. Expression levels of FN in treating M-HeLa cells with siRNA targeting FN (A), β1 integrin in treating M-HeLa cells with siRNA targeting β1 integrin (B) and EGFR in treating M-HeLa cells with siRNA targeting EGFR (C) were determined using real-time RT-PCR. Control-M-HeLa cells transfected with siRNA containing scrambled nucleotide sequence. Values are expressed as mean ± S.D. (error bars). A difference was considered significant at the $p<0,05$ level.
